# Self-Assembled Monolayers of Molecular Conductors with Terpyridine-Metal Redox Switching Elements: A Combined AFM, STM and Electrochemical Study

**DOI:** 10.3390/molecules27238320

**Published:** 2022-11-29

**Authors:** Jana Kocábová, František Vavrek, Štěpánka Nováková Lachmanová, Jakub Šebera, Michal Valášek, Magdaléna Hromadová

**Affiliations:** 1 J. Heyrovský Institute of Physical Chemistry of the Czech Academy of Sciences, Dolejškova 3, 18223 Prague, Czech Republic; 2 Institute of Nanotechnology, Karlsruhe Institute of Technology, P.O. Box 3640, 76021 Karlsruhe, Germany

**Keywords:** self-assembled monolayer, redox switching, electron transfer, AFM, STM

## Abstract

Self-assembled monolayers (SAMs) of terpyridine-based transition metal (ruthenium and osmium) complexes, anchored to gold substrate via tripodal anchoring groups, have been investigated as possible redox switching elements for molecular electronics. An electrochemical study was complemented by atomic force microscopy (AFM) and scanning tunneling microscopy (STM) methods. STM was used for determination of the SAM conductance values, and computation of the attenuation factor β from tunneling current–distance curves. We have shown that SAMs of **Os-tripod** molecules contain larger adlayer structures compared with SAMs of **Ru-tripod** molecules, which are characterized by a large number of almost evenly distributed small islands. Furthermore, upon cyclic voltammetric experimentation, **Os-tripod** films rearrange to form a smaller number of even larger islands, reminiscent of the Ostwald ripening process. **Os-tripod** SAMs displayed a higher surface concentration of molecules and lower conductance compared with **Ru-tripod** SAMs. The attenuation factor of **Os-tripod** films changed dramatically, upon electrochemical cycling, to a higher value. These observations are in accordance with previously reported electron transfer kinetics studies.

## 1. Introduction

Transition metal complexes rank among the most promising building blocks for new electrochromic [1,2] and molecular electronic devices [3,4,5,6,7,8,9,10,11,12]. This work presents a combined electrochemical and scanning probe microscopy (AFM and STM) study of the adsorption properties of the osmium and ruthenium–terpyridine (tpy) complexes that are connected to the gold electrode surface via a tripodal anchor. Our previous work confirmed the advantages of such groups in the design of sturdy and well conducting molecular wires for molecular electronics [13,14]. The binding affinity of terpyridine ligand towards cations decreases in the order Ru(II) > Os(II) > Fe(II) > Zn(II) > Cd(II). Consequently, octahedral “closed-shell” [M(tpy)_2_]^2+^ complexes of ruthenium, osmium and iron cations are the most suitable building blocks for the design of supramolecular architectures suitable for molecular electronic devices [6,9,15,16]. Furthermore, the electrically gated manipulation of spin states (spintronic devices) has been demonstrated in Mn(II)-containing terpyridine–metal complexes [5].

Current-voltage characteristics of molecular wires, based on metal–organic frameworks, have been studied previously by current sensing atomic force microscopy [17] and STM [18] techniques. Metal–organic wire systems also include bis(terpyridine)metal wires [6,7,19,20,21,22,23] with reported attenuation factor in the range β = 0.07−0.001 Å^−1^. Based on this low conductance attenuation ability, bis(terpyridine)metal wires are excellent candidates for long-distance charge transport that surpass even the charge transport characteristics reported for oligo-porphyrin molecular wires [18,24,25,26,27]. 

Self-assembled monolayers (SAMs) of ruthenium and osmium–bis-terpyridine complexes, with pendant 4-pyridyl substituent serving as an anchoring group, were studied in the work of Figgemeier et al. [28]. These authors used STM and electrochemistry for the characterization of SAMs on a Pt electrode. Higher surface coverage was found for the osmium complex compared with ruthenium one; whereas, Frumkin isotherm was utilized to account for the repulsive interactions within the monolayer. Electrochemical behavior in different solvents was explained by different solvents’ ability to screen charges according to its polarity. These authors also calculated the interaction energies between molecules within the monolayers. They found higher values for ruthenium complexes compared with their osmium analogues, which is consistent with the higher repulsive interactions observed for ruthenium complexes.

**Figure 1:**
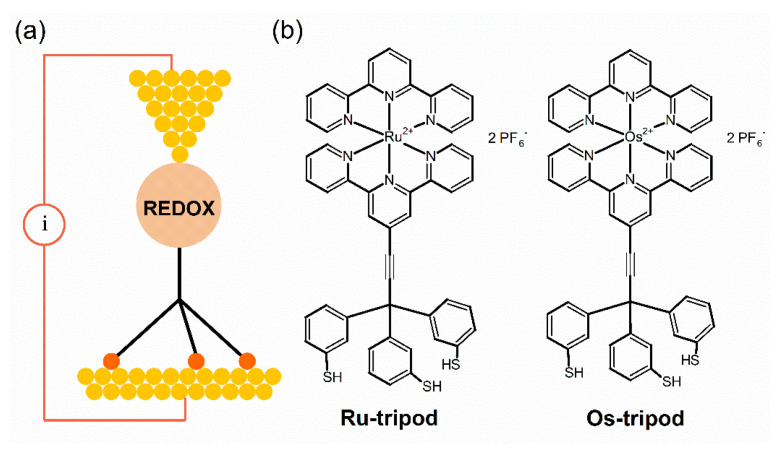
(**a**) Schematic arrangement of molecule with [M(tpy)_2_]^2+^ redox center in STM experiment; (**b**) chemical structure of **Ru-tripod** and **Os-tripod** molecules.

The present work studies the electrochemical and charge transport properties of SAMs containing molecules that can be connected to gold electrode via three thiolate anchors, as seen in Figure 1. Such tripodal arrangement should provide better geometry [13,14,29,30,31,32] and enhanced electronic coupling between the electrode and redox switching element [33]. In our previous work [34], we reported that molecules shown in Figure 1 form SAMs with higher surface coverage for molecule **Os-tripod** compared with **Ru-tripod,** with less pronounced mutual interaction between redox centers. Interestingly, surface coverage was higher than that reported by Figgemeier et al. [28] for pyridine-anchored molecules, confirming favorable upright orientation of redox centers due to the presence of covalently bonded tripodal pedestals. We also reported electron transfer (ET) rate constants for these SAMs. They were obtained by three independent electrochemical methods, providing the value of 1.4 × 10^3^ s^−1^ for **Os-tripod** SAM and 1.6 × 10^3^ s^−1^ for **Ru-tripod** SAM. Thus, ET was slightly faster within the **Ru-tripod** SAMs.

## 2. Results and Discussion

**Figure 2:**
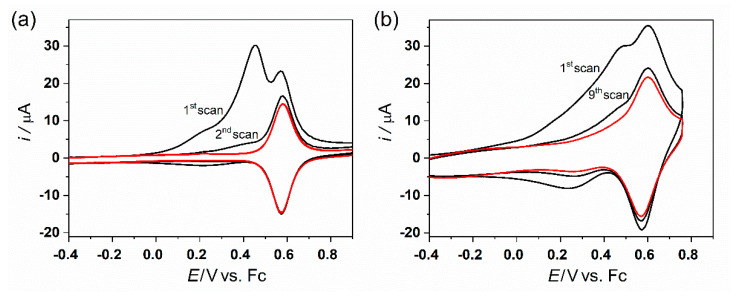
Cyclic voltammogram for **Os-tripod** SAM on: (**a**) Au bead polycrystalline electrode in 0.5 M TBAPF_6_ in acetonitrile, electrode area 0.267 cm^2^; (**b**) Au(111) on mica electrode in 0.1 M TBAPF_6_ in acetonitrile, immersed geom. area approx. 0.55 cm^2^. Scan rate was 0.1 V s^−1^. Red color indicates final voltammogram.

**Figure 3:**
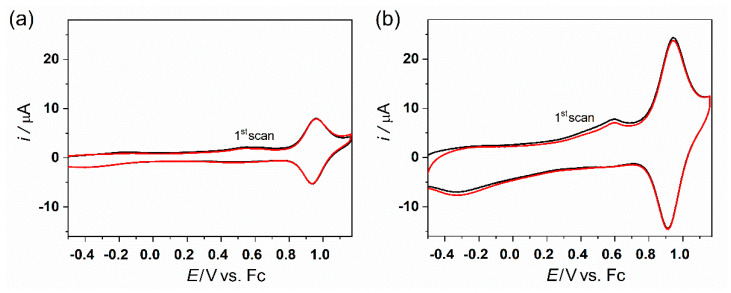
Cyclic voltammogram for **Ru-tripod** SAM on: (**a**) Au bead polycrystalline electrode in 0.5 M TBAPF_6_ in acetonitrile, electrode area 0.267 cm^2^; (**b**) Au(111) on mica electrode in 0.1 M TBAPF_6_ in acetonitrile, immersed geom. area approx. 0.55 cm^2^. Scan rate was 0.1 V s^−1^.

SAMs of molecules shown in Figure 1 were prepared on a large monocrystalline Au(111) on mica electrode and studied using a combination of cyclic voltammetry and scanning probe techniques. Figure 2 shows a typical cyclic voltammogram (CV) obtained in the acetonitrile solvent, using a tetrabutylammonium hexafluorophosphate (TBAPF_6_) supporting electrolyte for the oxidation of the **Os-tripod** SAM, chemisorbed either on a polycrystalline gold bead (**a**) or a monocrystalline Au(111) on mica (**b**) electrode, for comparison. In both cases, the electrochemical behavior of the **Os-tripod** SAM was similar. Namely, during the first potential scan in the positive potential direction, two distinct current peaks were observed; the first peak disappeared upon repeated electrode potential cycling between the negative and positive potential directions, leading to a final steady-state CV, shown in the red color. It has characteristics typical of a 1-electron reversible surface-confined redox system with repulsive interactions between the individual redox-active moieties, meaning that the full width at half maximum of the peak current is larger than 90.6 mV, which is theoretically predicted for non-interacting redox centers in the adsorbed state [34]. Figure 3 shows the same type of the voltammetric experiment for the **Ru-tripod** SAM. CV shows only one oxidation and one reduction peak, independent of the type of the gold electrode substrate or the number of the potential cycles needed to reach the final steady-state signal. Thus, the main difference between these two films seems to be the molecular arrangement within the SAM structure. The full electrochemical characterization of the final voltammograms on the gold bead electrode has been provided in our previous publication [34]. In summary, the surface concentration of the **Os-tripod** film was found to be higher than that of the **Ru-tripod** SAM; whereas, from the comparison of the full width at half maximum of the peak current, it was concluded that the repulsion interactions are more pronounced in the **Ru-tripod** SAMs. This difference in the degree of repulsive interactions was used to explain the higher surface concentration of **Os-tripod** molecules in the compact SAM, compared with the **Ru-tripod** SAM [34].

Previously obtained results, as well as the present measurements on Au(111) electrode surface, are in very good agreement with experimental observations reported by Figgemeier et al. [28] for ruthenium and osmium–terpyridine complexes anchored to the electrode by only one anchoring group; the only difference being a much higher surface concentration of tripodal molecules reported in this work compared with monopodal molecules. The saturation surface concentration of monopodal ruthenium and osmium complexes on the Pt electrode surface was reported to be (2.5 ± 0.2) × 10^−11^ and (3.3 ± 0.2) × 10^−11^ mol cm^−2^, respectively. The same procedure, using the charge under the oxidation and reduction peaks, led to the value of (4.0 ± 0.2) × 10^−10^ and (4.5 ± 0.2) × 10^−10^ mol cm^−2^ for the **Ru-tripod** and **Os-tripod** SAMs on the polycrystalline gold bead electrode [34]. The difference in the saturation surface concentration between this work and that of Figgemeier et al. [28] stems from the use of different anchoring groups. Whereas the aforementioned [28] authors used a conventional pyridine anchor, we used a tripodal thiol-based anchoring group. In the former case, the bond between the nitrogen and Pt is not particularly strong, thus allowing the movement of the redox centers on the electrode surface and enabling the repulsive interactions between the individual molecules to dictate the surface concentration, leading to a rather loose packing. In the case of a thiolate–gold bond (three bonds per one redox unit), the redox centers are less likely to move away from each other on the electrode surface due to their repulsive interactions (compare CVs in Figure 2 and Figure 3). Our tripodal anchors thus dictate the packing of the molecules on the electrode surface, and at the same time keep the redox units more or less perpendicular to the electrode surface. This arrangement allows more individual molecules to be packed within the SAM. Our previous studies of SAMs using similar tripodal anchoring groups [14] reported a surface concentration of 5.2 × 10^−10^ mol cm^−2^ for compact monolayers, which is close to the values reported in this work.

**Figure 4:**
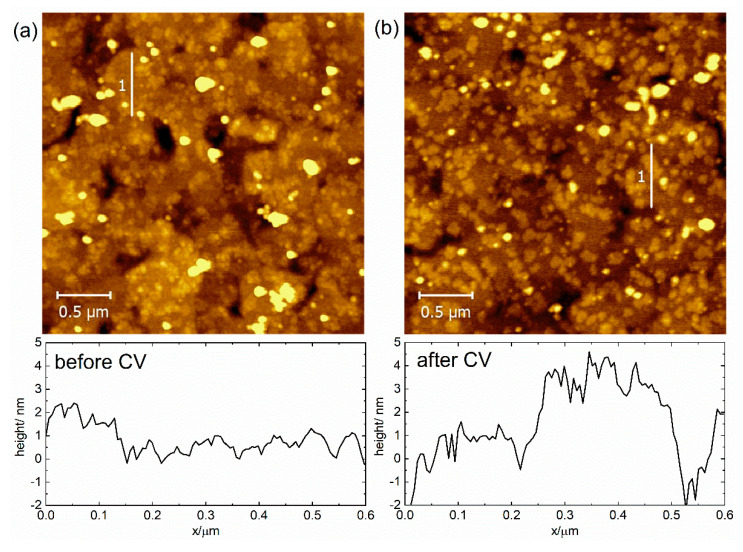
AFM topography image of 3 × 3 μm^2^ and graph of height profile along line 1 (upwards) for **Os-tripod** SAM on Au(111): (**a**) before; (**b**) after cyclic voltammetry experiment in 0.1 M TBAPF_6_ in acetonitrile at scan rate 0.1 V s^−1^.

**Figure 5:**
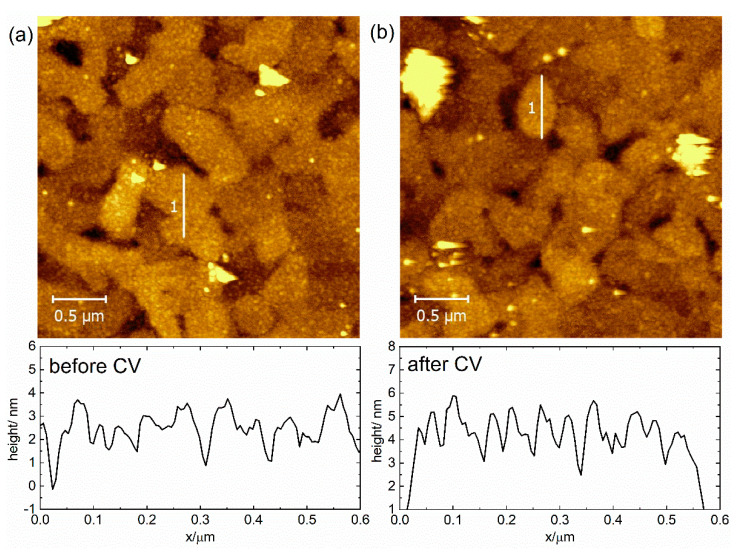
AFM topography image 3 × 3 μm^2^ and graph of height profile along line 1 (upwards) for **Ru-tripod** SAM on Au(111): (**a**) before; (**b**) after cyclic voltammetry experiment in 0.1 M TBAPF_6_ in acetonitrile at scan rate 0.1 V s^−1^.

After we confirmed similar CV behavior for both polycrystalline and monocrystalline gold electrodes (see Figure 2 and Figure 3), we subjected the SAMs chemisorbed on the monocrystalline Au(111) to atomic force microscopy (AFM) studies. The AFM method is based on surface imaging at the preset constant force between the tip and the SAM surface. AFM was used with the tapping mode regime to avoid damage to the SAM. Corresponding AFM topography images for the **Os-tripod** SAM are shown in Figure 4, and for the **Ru-tripod** SAM in Figure 5. Each figure provides two images. The left image (**a**) was obtained from the SAM surface directly after its preparation. The right image (**b**) was taken from the part of the surface that was subjected to the CV experiment (red curves) shown in either Figure 2b for the **Os-tripod** SAM or in Figure 3b for the **Ru-tripod** SAM, respectively.

All topography images in Figure 4 and Figure 5 represent a surface area of 3 × 3 μm^2^. The main difference between the **Os-tripod** and **Ru-tripod** SAMs concerns the overall surface structure appearance. Even though we are not able to achieve molecular level resolution or confirm the existence or absence of more than one layer of molecules on the electrode surface, we know that the surface coverages, based on the experimentally-determined surface concentrations on the bead electrodes, must be very close to one (the exact value would, of course, depend on the molecular packing model used). Nevertheless, comparison of the AFM images of the **Os-tripod** and **Ru-tripod** SAMs before the electrochemical experiment indicates the presence of a higher number of small and evenly distributed islands of **Ru-tripod** molecules, compared with the lower number of larger ones for **Os-tripod** molecules. Height profiles along the selected lines in the AFM images confirm this statement. Whereas, for the **Os-tripod** SAM (Figure 4a), height changes of less than 1 nm can be observed in the AFM image, much larger differences in the height profile—spaced at much shorter lateral distances—can be obtained for **Ru-tripod** SAM (see Figure 5a). What is more important is the effect of the electrode potential cycling on the topography of the compact films. In this work, we have been able to show that the surface topography does change for the **Os-tripod** SAM; meanwhile, it stands virtually the same in the case of the **Ru-tripod** films (compare Figure 4 and Figure 5). One can argue that in the case of the **Ru-tripod** film, the islands are even more equally distributed, but the overall characteristics of the layer stay the same. On the other hand, the **Os-tripod** SAM, after the potential cycling leads to the formation of larger adlayer structures (similar to Ostwald ripening process). This process can be considered a convincing demonstration of the less repulsive nature of mutual interactions between the individual redox centers in **Os-tripod** SAMs compared with **Ru-tripod** ones. At this point, we should note that AFM images were obtained ex situ, in the air, before and after the electrochemical experiments shown in Figure 2b and Figure 3b.

**Figure 6:**
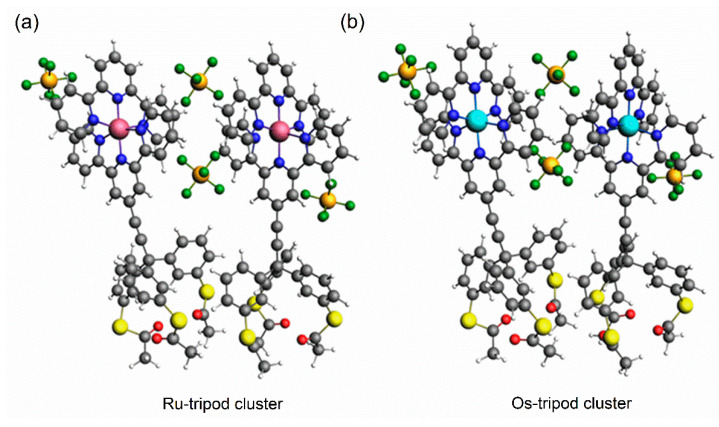
Geometry-optimized cluster of (**a**) **Ru-tripod**; and (**b**) **Os-tripod** molecules containing acetyl-protected thiolates.

We attempted to provide more insight into the observed experimental differences in CV and AFM measurements by using quantum chemical calculations of the interaction energies E_int_ between two molecules of either **Ru-tripod** or **Os-tripod** complexes, in the form of acetyl-protected thiols. Calculations were performed for neutral clusters including two PF_6_^-^ counterions per molecule in vacuo. Final geometry-optimized cluster structures are shown in Figure 6. Computational details are given in the Section 3.5.

The E_int_ was calculated using the equation E_cluster_ − (E_1_ + E_2_) where E_cluster_ is the energy of the cluster of two **Ru-tripod** or **Os-tripod** molecules, and E_1_ and E_2_ are the energies of individual molecules. 

The value of E_int_ calculated for the **Ru-tripod** cluster is −33.9 kcal/mol, and for the **Os-tripod** cluster is −37.4 kcal/mol. This difference indicates that the formation of **Os-tripod** clusters is more likely than the formation of **Ru-tripod** clusters, which is in accordance with our AFM observations. These results are further supported by different distances between Os atoms (9.98 Å) and Ru atoms (10.69 Å) in the calculated cluster geometries. These calculations also provide the rationale for greater repulsive interactions between individual **Ru-tripod** molecules in self-assembled monolayers observed in the cyclic voltammetric studies. Indeed, the full width at half maximum of the CV peak for the Os-tripod SAM is 103 mV (see Figure 2); whereas, it is 138 mV for the CV peak of the Ru-tripod SAM (see Figure 3). The counterions also play an important role in the stabilization of the self-assembled monolayers. We have observed that the average distance between the central Os atom and nearest neighbor P atoms (of hexafluoro phosphate anions) is 5.63 Å, whereas it is 6.10 Å for the central Ru atom and nearest neighbor P atoms. Even though our E_int_ calculated values cannot be directly compared with the interaction energies obtained by Figgemeier et al. [28] for their molecules, we observed the same trend. Namely, that the interaction energy of Os-containing complexes in the SAM has a higher negative value compared with Ru-containing complexes. These authors employed a statistical mechanical treatment in which they had to assume the number of nearest neighbors within the monolayer.

**Figure 7:**
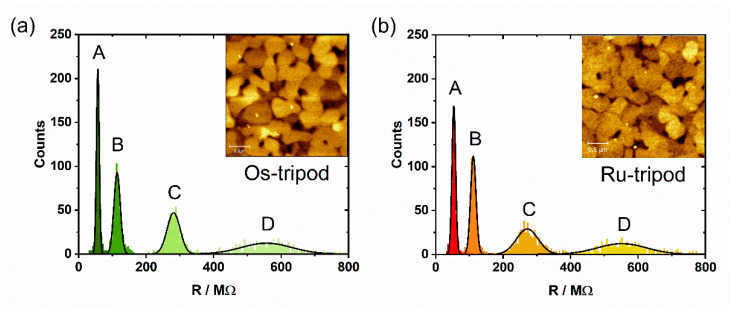
1D histograms of resistance values for: (**a**) **Os-tripod** SAM; (**b**) **Ru-tripod** SAM. Data obtained from 500 I-V curves measured at different setpoint currents 1 nA (A), 0.5 nA (B), 0.2 nA (C), 0.1 nA (D) and constant bias voltage 0.05 V, bin size was 5 MΩ. Black lines show the best Gaussian fit. AFM topography images of the corresponding samples are shown in the inset.

Knowing the state of the SAMs, and employing the AFM imaging, we utilized STM methodology [35,36] to obtain the conductance *G* of both Au|SAM assemblies. In this work, we characterized SAMs of **Os-tripod** and **Ru-tripod** using a series of current–voltage (I–V) and current–distance (I–z) measurements. The I–V characteristics of molecular wires [37] provided the value of conductance *G* from their slope—at low bias voltage V—in the so-called ohmic regime. Values reported here represent *G,* calculated as ∆I/∆V within the ± 0.01 V interval. In this work, we measured the conductance values at four constant distances between the gold substrate and tip, which were accomplished by applying four different setpoint currents, namely 0.1, 0.2, 0.5 nA and 1 nA. A statistically-significant number of I–V curves were analyzed at each setpoint current to provide the resistance R = 1/*G* for the Au|SAM system. These measurements were taken using freshly prepared **Os-tripod** and **Ru-tripod** SAMs. Figure 7 shows the resistance histograms for Au|**Os-tripod** and Au|**Ru-tripod** SAM systems, obtained by the evaluation of 500 I–V curves at each setpoint current. The inset shows the AFM image of the actual sample being analyzed. In general, the lower the setpoint current (and the further the distance of the gold tip from the SAM surface) the larger the Gaussian peak width, and the wider the spread of resistance values. In addition, the shorter the distance between the Au|SAM surface and the gold tip, the more closely the I–V curves should represent the conductance of the SAM, without a significant contribution from the through-space (through-air) tunneling.

Table 1 summarizes the most probable resistance (conductance) values of the Au|SAM systems, obtained from the histograms in Figure 7, at four distances from the SAM surface. One can see that the obtained resistance (conductance) of the **Os-tripod** SAM is always higher (lower) than that of the **Ru-tripod** one. This would mean that the osmium-based monolayer as prepared is less conducting, based purely on the tunneling current measurements. Table 1 clearly shows that the conductance decays exponentially with the distance, since *G* values obtained from I–V curves scale linearly with the setpoint currents used. Assuming the simple Simmons model for temperature-independent non-resonant tunneling [38,39,40] one can assign a constant effective tunneling barrier height to each Au|SAM system, which would be obviously lower for the **Ru-tripod** SAM. In this model, an electron with an effective mass *m* tunnels through a rectangular barrier of height ΔE to the interelectrode distance d. The current drops exponentially, providing I~exp(−βd), where the attenuation factor β has the form (2/ħ)$\sqrt{2m\Delta E}$.

**Figure 8:**
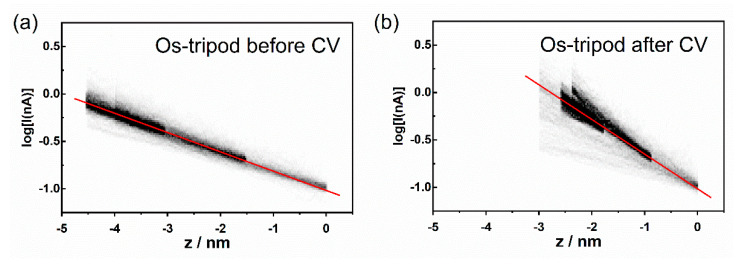
2D semilogarithmic histogram of tunneling current–distance (I–z) curves for the **Os-tripod** SAM on Au(111): (**a**) before; (**b**) after cyclic voltammetry experiment. Three sets of I–z curves were measured starting at setpoint currents of 0.1 nA, 0.2 nA and 0.4 nA. Approach rate was 1 nm s^−1^, bin size log(I) = 0.02, z = 0.02 nm. Red line represents the best least square fit of the most probable log(I) values as a function of z.

**Figure 9:**
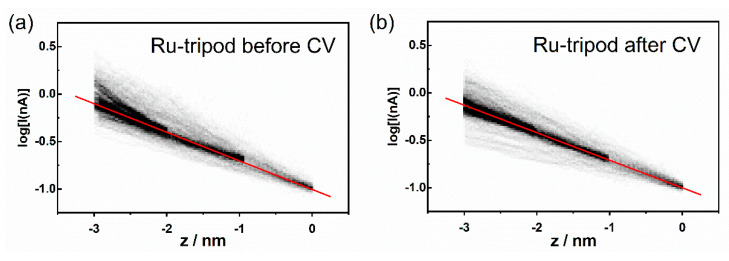
2D semilogarithmic histogram of tunneling current–distance (I–z) curves for the **Ru-tripod** SAM on Au(111): (**a**) before; (**b**) after cyclic voltammetry experiment. Three sets of I–z curves were measured starting at setpoint currents of 0.1 nA, 0.2 nA and 0.4 nA. Approach rate was 1 nm s^−1^, bin size log(I) = 0.02, z = 0.02 nm. Red line represents the best least square fit of the most probable log(I) values as a function of z.

At this point, we measured the current–distance (I–z) curves for both systems, from which one can obtain the effective tunneling barrier height directly through the evaluation of the attenuation factor β. The measurements were taken at three distances of the gold tip from the Au|SAM system, which were realized by the preset constant value of the setpoint current indicated in the figure caption. Each histogram in Figure 8 and Figure 9 represents 300 current–distance experimental curves. The darkest areas indicate the most frequently observed [log(I);z] data pairs. Data were further analyzed to obtain the most probable linear dependence between the log(I) and z values. This analysis required us first to determine the most probable [log(I);z] data pair from the histograms in Figure 8 and Figure 9. This was accomplished by finding the most probable z value from the best Gaussian fit of the dominant z peak (one-dimensional z histogram), observed at constant log(I) value, i.e., along the horizontal line at any preselected log(I) value. After the most probable [log(I);z] data pairs were obtained, they were used to calculate the most probable linear dependence between log(I) and z (see red line in Figure 8 and Figure 9). The slopes of the best linear, least square fits to these red lines are summarized in Table 2.

Figure 8 shows the 2D semilogarithmic histogram of the I–z curves for the **Os-tripod** SAM before and after electrochemical experiment. Figure 9 shows the same for the **Ru-tripod** SAM. Although there is a slope change in the log(I)-z histogram for osmium-based films before and after the electrochemical experiment, no such change was observed for **Ru-tripod** SAMs. This result is in accordance with the AFM observation of the structural change of the SAM in the case of the **Os-tripod** film. The actual values of the attenuation factors β are summarized in Table 2. They represent the slopes of the red lines in Figure 8 and Figure 9. The β attenuation factor of the **Ru-tripod** SAM changes only slightly from 0.693 nm^−1^ to 0.668 nm^−1^; however, there is a significant change in the β value for the **Os-tripod** film upon cyclic voltammetric experiment. The β value, corresponding to the **Os-tripod** SAM structure shown in Figure 4b, is 0.843 ± 0.021 nm^−1^, which is much higher than 0.668 ± 0.007 nm^−1^ obtained for the **Ru-tripod** SAM (see corresponding SAM structure in Figure 5b). This experimental result means that the effective barrier height for charge transport should be much smaller for the **Ru-tripod** SAM compared with the **Os-tripod** SAM structure.

Comparing the results from STM characterization using both I–V and I–z measurements, one can state that based on the I–V curves, the Au|SAM structure based on **Os-tripod** molecules has lower conductance, while the current (conductance) attenuation factor changes dramatically upon cycling between two redox states of this transition metal complex. In any case, the lower conductance value for the **Os-tripod** SAM observed in this work is in accordance with previously observed differences in the electron transfer rate constants of these layers, pointing to the energetically more demanding but faster process, in the case of ET in **Ru-tripod** SAMs.

## 3. Materials and Methods

### 3.1. Chemicals and Materials

Molecules for the preparation of the **Os-tripod** and **Ru-tripod** SAMs were prepared according to previously described procedures in the form of thiol-protecting acetates [13,34,41]. The following chemicals were used as received: gold wire, 0.25 mm in diameter, 99.99+% purity, Goodfellow; ferrocene, 98%, Fluka, Switzerland; ethanol, 99.8%, molecular biology grade, Applichem GmbH, Germany and p.a. Penta, Czech Republic; triethylamine, ≥99.5%, BioUltra, Sigma-Aldrich, Belgium; nitric acid, 65%, p. a. Lach-Ner, Czech Republic; sulfuric acid, 96%, p. a. Lach-Ner, Czech Republic; hydrogen peroxide, 30%, p. a. unstabilized, Lach-Ner, Czech Republic; argon gas, 99.998%, Messer, Czech Republic. Acetonitrile, 99.8%, anhydrous, Sigma-Aldrich, Germany was dried with activated sieves size 0.3 nm, Lachema, Czech Republic. Further treatment to remove water residue was performed by alumina powder activated in the oven at 200 °C. Tetrabutylammonium hexafluorophosphate, ≥99%, p. a., for electrochemical use, Sigma-Aldrich, Switzerland was dried in the oven at 80 °C before use.

All glassware, PTFE chambers and Kalrez O-rings for SAM preparation, electrochemistry and STM characterization were cleaned by boiling in 25% nitric acid, which was followed by repeated boiling in deionized water of resistivity 18.2 MΩ cm and maximum TOC 3 ppb (Milli-Q Integral 5 water purification system, Merck Millipore, Saint-Quentin Fallavier, France).

### 3.2. SAM Preparation

Au bead electrodes (area 0.267 cm^2^, determined experimentally in 0.1 M sulfuric acid [42]) were cleaned in freshly prepared Piranha solution (sulfuric acid and peroxide in the *v/v* ratio 3:1) and flame-annealed with a butane torch before the SAM deposition. CAUTION: Piranha solution must be handled with care! Au(111) on mica (area 1 × 1.1 cm^2^, Keysight technologies, Wokingham, UK) electrodes were flame-annealed with the butane torch and cooled down under the argon atmosphere before SAM formation. Glass weighing bottles were filled with 3.3 mL of 5 × 10^−5^ M ethanolic solution of either **Os-tripod** or **Ru-tripod** acetate-protected molecules. Triethylamine deprotecting agent (0.33 mL) was added immediately after immersion of the gold electrodes. Bottles were de-aerated with argon gas and closed. They were kept in closed PTFE chambers at 60 °C for 19 h. Subsequently, each substrate was washed copiously—at least five times—with absolute ethanol and dried in a stream of Ar gas. Au bead electrodes were used for comparative CV measurements and Au(111) electrodes were used for cyclic voltammetry, AFM and STM measurements.

### 3.3. Electrochemistry

Cyclic voltammetry measurements were performed in an all glass, electrochemical three-electrode cell. Gold electrodes served as the working electrode, Pt wire as the auxiliary electrode and a Ag|AgCl|1 M LiCl electrode served as the reference electrode. The latter was separated from the main compartment by a salt bridge via double-fritted junction. Oxygen was removed by a stream of Ar gas, which blanketed solution throughout the experiment. Ferrocene was used as an internal standard. All CV experiments were performed using potentiostat PGSTAT30 (Metrohm, Herisau, Switzerland) equipped with an FRA2 impedance module. Positive feedback iR compensation was used.

### 3.4. AFM and STM Measurements

AFM imaging and STM spectroscopy of SAMs in the air were performed using Agilent 5500 Scanning Probe Microscope (Agilent Technologies, Palo Alto, CA, USA). Tapping mode AFM was used for obtaining topography images, using AAC cantilevers of the nominal resonant frequency 190 kHz and nominal force constant 48 N/m. STM measurements were performed with electrochemically-etched gold tips, according to the etching procedure reported elsewhere [43]. AFM images were examined using SPM data visualization and analysis software Gwyddion 2.41 (Czech Metrology Institute, Jihlava, Czech Republic) [44]. All shown AFM images were plane corrected. Each current–voltage I–V curve was obtained at constant distance between STM tip and substrate (defined by the selected setpoint current value and bias voltage 50 mV). During I–V curve measurements, the substrate voltage was swept between −0.2 V and 0.2 V at one of the two potential sweep duration times, 0.48 s or 1.04 s. For each STM tip–substrate distance, 500 curves were collected, each containing 2000 data points. Resistance values were obtained from the slopes of the individual I–V curves, within the potential range between −0.01 V and 0.01 V. These measurements were obtained from SAM samples that were subjected to topographic AFM imaging only. Current–distance (I–z) curves were initiated at three different distances from the electrode (100 curves for each starting position), and can be considered as the STM tip approach curves. The starting z piezo position was determined by selected sample bias (50 mV) and setpoint current value, and maintained by a feedback loop between each I–z curve measurement. The approach rate was 1 nm s^−1^. Data at three setpoint currents (0.1, 0.2 and 0.4 nA) were grouped together and a 2D histogram of [log(I);z] pairs was computed using the bin size of log(I) = 0.02 and z = 0.02 nm. Further analysis required construction of 1D histograms of z values at preselected log(I) (horizontal lines in 2D histograms), followed by the Gaussian fit of the dominant z peak. These thus-obtained points (Gaussian maxima) were fitted to a straight line (see red lines in Figure 8 and Figure 9). STM measurements were performed on samples before as well as after CV measurements.

### 3.5. Theoretical Calculations

Clusters of **Ru-tripod** and **Os-tripod** complexes (see Figure 6) were formed from two **Ru-tripod** and **Os-tripod** molecules. The geometry-optimized structures of individual **Ru-tripod** and **Os-tripod** molecules are not shown.

Firstly, the **Ru-tripod** complex was geometry optimized by B3LYP [45] functional applied within the density functional theory (DFT). DFT calculation included a D3 dispersion coefficient [46]. The 6-31G(d) basis set for S, N, H, O atoms [47,48,49,50,51] and the LANL2DZ [52] basis set (including relevant relativistic pseudopotential) for Ru atoms were used. The polarizable continuum model (PCM), describing acetonitrile as solvent, was also used [53]. This geometry-optimized task was performed by Gaussian quantum chemistry program version 09 [54]. This complex was modelled with positive charge 2 e. The vibration analysis was performed within linear harmonic approximation. No imaginary frequencies were obtained by this vibrational analysis. Secondly, on the basis of the geometry-optimized structure of the **Ru-tripod** complex, the new complex of **Ru-tripod** cation interacting with two PF_6_^−^ counterions was prepared. The structure of this molecule was geometry optimized (in vacuo) by using program MOPAC [55] as implemented in the graphical user interface of ADF program version 2017 [56]. For the geometry optimization step, the semiempirical PM6-D3H4 method was used. The calculations performed by PM6-D3H4 method using PCM approach are not implemented in the GUI of the ADF program used. Therefore, these calculations were performed in an in vacuo environment. The PM6-D3H4 quantum chemistry method included corrections to hydrogen bonding and dispersion interactions [57]. The resulting **Ru-tripod** structure was used for geometry optimization of the **Ru-tripod** cluster (consisting of two **Ru-tripod** molecules). The electronic energy of the geometry-optimized cluster was used as the value of E_cluster_ energy for calculation of the E_int_ energy. Finally, the individual **Ru-tripod** molecules were geometry optimized using PM6-D3H4 method, starting from the geometry of individual molecules in the geometry-optimized **Ru-tripod** cluster in vacuo. The resulting structures of molecules 1 and 2, and their electronic energies E_1_ and E_2_ were used for calculation of the E_int_ energy according to the equation E_int_ = E_cluster_ − (E_1_ + E_2_). The initial structure of the **Os-tripod** cluster (consisting of two **Os-tripod** molecules) was created through the substitution of Ru atoms with Os atoms. Then, the **Os-tripod** cluster was geometry optimized using PM6-D3H4 method. The E_int_ was calculated using the same procedure as used for the **Ru-tripod** cluster mentioned above.

## 4. Conclusions

A combined electrochemical and scanning probe microscopy study of the conductance properties of SAMs of two transition metal complexes, with a rigid tripodal anchoring scaffold to the conducting substrate, has been discussed. The AFM characterization of SAMs revealed the differences in the overall structural details between the **Ru-tripod** and **Os-tripod** SAMs, in accordance with different degrees of repulsive interactions as observed by cyclic voltammetry. A quantum-chemical computation of the interaction energies between molecules also supports this observation. The conductance properties of each film were studied by scanning tunneling spectroscopy, assuming a simple Simmons model for non-resonant tunneling in the Au|SAMs. The conductance values at different distances from the SAM surface were systematically lower for **Os-tripod** molecules, indicating better conductance of **Ru-tripod** SAMs. The later SAMs are characterized by a lower surface concentration of **Ru-tripod** molecules on the electrode surface, with more evenly distributed molecules. Both AFM and STM characterization showed changes in the structural and conducting characteristics of **Os-tripod** SAMs upon cyclic voltammetric experiment, i.e., upon the redox switching of the Os^2+/3+^ center in the transition metal complex. Interestingly, the conductance characteristics of these SAMs obtained in the non-resonant tunneling regime correlate with the electron transfer rate constants obtained for SAMs in a classical electrochemical experiment. Our results demonstrate the promising potential of tripodal scaffolds for the fabrication of molecular electronic devices profiting from the electrochemical features of the molecular layer, where such tripodal architectures enhance control and stability over the surface geometry, and thus improve the arrangement of the exposed functionality on metallic surfaces.

## Figures and Tables

**Table 1:** Resistance (MΩ) and conductance (nS) values for Au|SAM systems obtained from I–V curves at different setpoint currents indicated, and at constant bias voltage of 0.05 V. Potential sweep rate was 0.38 V/s.

Setpoint Current *	0.1 nA	0.2 nA	0.5 nA	1.0 nA
**Ru-tripod** SAM	552.0 ± 73.1 MΩ 1.81 ± 0.24 nS	271.8 ± 31.6 MΩ 3.68 ± 0.43 nS	111.7 ± 8.2 MΩ 8.95 ± 0.65 nS	53.9 ± 5.5 MΩ 18.6 ± 1.9 nS
**Os-tripod** SAM	557.0 ± 74.6 MΩ 1.79 ± 0.24 nS	281.6 ± 19.3 MΩ 3.55 ± 0.24 nS	114.0 ± 9.5 MΩ 8.77 ± 0.73 nS	57.0 ± 4.3 MΩ 17.5 ± 1.3 nS

* Resistance (conductance) values obtained from Gaussian peak fits of the histograms in Figure 7.

**Table 2:** Attenuation factor β for Au|SAM systems obtained from tunneling current–distance curves by statistical analysis.

System	Attenuation Factor	
	Before CV	After CV
**Ru-tripod** SAM	(0.693 ± 0.012) nm^−1^	(0.668 ± 0.007) nm^−1^
**Os-tripod** SAM	(0.468 ± 0.005) nm^−1^	(0.843 ± 0.021) nm^−1^

## Data Availability

All original data files are available upon request from the institutional repository.

## Sample Availability

Samples of the compounds are not available from the authors.
